# Activation of gelatinases in permanent human teeth after different experimental radiotherapy protocols

**DOI:** 10.1590/0103-6440202305542

**Published:** 2023-12-22

**Authors:** Paula Iáddia Zarpellon Barbosa, Ricardo Barbosa Lima, Lucas Masaru Marubayashi, Harley Francisco de Oliveira, Raquel Assed Bezerra da Silva, Paulo Nelson-Filho, Maya Fernanda Manfrin Arnez, Francisco Wanderley Garcia de Paula-Silva, Alexandra Mussolino de Queiroz

**Affiliations:** 1 Graduate Program in Pediatric Dentistry, School of Dentistry of Ribeirão Preto, University of São Paulo(FORP/USP), Ribeirão Preto, São Paulo, Brazil; 2 Department of Internal Medicine, Ribeirão Preto School of Medicine, University of São Paulo (FMRP/USP), Ribeirão Preto, São Paulo, Brazil; 3 Department of Pediatric Clinics, School of Dentistry of Ribeirão Preto, University of São Paulo(FORP/USP), Ribeirão Preto, São Paulo, Brazil

**Keywords:** head and neck neoplasms, radiotherapy, radiation dose hypofractionation, gelatinases, tooth diseases.

## Abstract

The objective of this study was to compare the activation of gelatinases in dentin-enamel junction (DEJ) and underlying dentin of permanent teeth after experimental radiotherapy in conventional and hypofractionated modalities. Newly extracted third molars (*n* = 15) were divided into three experimental radiotherapy groups: control, conventional (CR), and hypofractionated (HR) (*n* = 5 per group). After *in vitro* exposure to ionizing radiation, following standardized protocols for each modality, a gelatinous substrate was incubated on the tooth slices (*n* = 10 per group). Activation of gelatinases was measured by *in situ* zymography, expressed in arbitrary fluorescence units (mm^2^) from three tooth regions: cervical, cuspal, and pit. Fluorescence intensity was compared among radiotherapy protocols and tooth regions in each protocol, considering a significance level of 5%. Considering all tooth regions, the fluorescence intensity of the CR group was higher than the HR and control groups, both in DEJ and underlying dentin (*p* <0.001). In addition, the fluorescence intensity was higher in underlying dentin when compared to DEJ in all groups (*p* <0.001). Considering each tooth region, a statistically significant difference between CR and HR was only observed in the pit region of underlying dentin (*p* <0.001). Significant and positive correlations between fluorescence intensities in DEJ and underlying dentin were also observed (*p* <0.001). Experimental radiotherapy influenced the activation of gelatinases, as well as exposure to the conventional protocol can trigger a higher activation of gelatinases when compared to hypofractionated, both in DEJ and underlying dentin.

## Resumo

O objetivo deste estudo foi comparar a ativação de gelatinases na junção dentina-esmalte (DEJ) e na dentina subjacente de dentes permanentes após a radioterapia experimental nas modalidades convencional e hipofracionada. Os terceiros molares recém-extraídos (n = 15) foram divididos em três grupos de radioterapia experimental: controle, convencional (CR) e hipofracionada (HR) (n = 5 por grupo). Após a exposição *in vitro* à radiação ionizante, seguindo protocolos padronizados para cada modalidade, um substrato gelatinoso foi incubado nas fatias de dente (n = 10 por grupo). A ativação das gelatinases foi medida por zimografia in situ, expressa em unidades arbitrárias de fluorescência (mm^2^) de três regiões do dente: cervical, cúspide e fossa. A intensidade da fluorescência foi comparada entre os protocolos de radioterapia e as regiões do dente em cada protocolo, considerando um nível de significância de 5%. Considerando todas as regiões do dente, a intensidade de fluorescência do grupo CR foi maior do que a dos grupos HR e controle, tanto no DEJ quanto na dentina subjacente (*p* <0,001). Além disso, a intensidade da fluorescência foi maior na dentina subjacente quando comparada à DEJ em todos os grupos (*p* <0,001). Considerando cada região do dente, uma diferença estatisticamente significativa entre CR e HR foi observada apenas na região da fossa da dentina subjacente (*p* <0,001). Também foram observadas correlações significativas e positivas entre as intensidades de fluorescência no DEJ e na dentina subjacente (*p* <0,001). A radioterapia experimental influenciou a ativação das gelatinases, assim como a exposição ao protocolo convencional pode desencadear uma maior ativação das gelatinases quando comparada ao hipofracionamento, tanto no DEJ quanto na dentina subjacente.

## Introduction

Radiotherapy is a relevant treatment modality for patients with head and neck cancer (HNC), positively interfering in reducing the risk of death, and can be combined with other approaches to enhance the HNC therapy, such as surgery and/or chemotherapy^1^. However, although it is relevant, exposure to ionizing radiation has side effects on adjacent structures in the irradiated region, such as the oral cavity (*e*.*g*., teeth) when considering HNC radiotherapy. Exploring this theme, the activation of gelatinases (matrix metalloproteinases 2 and 9; MMP-2 and -9, respectively) in primary and permanent teeth exposed to experimental conventional radiotherapy protocol were investigated. Primary and permanent molars were used to make dental fragments, submitting part of them to ionizing radiation to simulate a radiotherapy exposure (2Gy fractions for five consecutive days, until reaching a cumulative dose of 60Gy). Then, using *in situ* zymography, an increased activity of gelatinases was observed in the dentin-enamel junction (DEJ), demonstrating a possible impact of this exposure on the degradation of the organic matrix^2^ ^,^ ^3^.

Understanding the activity of gelatinases after teeth exposure to radiotherapy protocols is valuable, considering that MMPs are significant mediators in the degradation of the organic matrix in dental hard tissues, which raised hypotheses about its role in the side effects triggered by ionizing radiation on teeth, such as radiation-related caries (RCC)^4^ ^,^ ^5^. Indeed, HNC patients exposed to radiotherapy often manifest a specific pattern of RRC, including physical and chemical changes in the tooth structure at the DEJ, which lead to enamel delamination^6^. In addition, due to changes in the proportion of proteins in the organic matrix of exposed teeth (reduced when compared to mineral content), it was possible to question whether ionizing radiation can induce these changes directly or indirectly (by activating MMPs)^7^.

The DEJ is an important anatomical tooth region to understand RRC patterns in HNC patients, considering its importance in properly maintaining the interface between enamel and underlying dentin, especially in the cervical region, where enamel delamination’s are often observed (an important event for susceptibility to dental caries). Among other factors, the integrity of tooth structure depends on the proper interface between the layers of enamel and dentin, as well as the associated organic matrix^6^ ^,^ ^7^, which leads us to question the impact of radiotherapy-induced activation of gelatinases on the degradation of organic matrix and DEJ integrity.

From a clinical-epidemiological point of view, when considering a worldwide incidence of more than 550.000 thousand HNC cases annually, as well as the exposure of more than 40% of patients to radiotherapy as a treatment modality^8^, the side effects become even more relevant. RRC is one of the main adverse effects related to radiotherapy and dental hard tissues, affecting up to 25% of the cancer patients exposed^6^. However, there is still a lack of information to understand radiotherapy as an independent risk factor for changes in DEJ after ionizing radiation exposure^8^.

Radiotherapy showed a significant impact on the management of head and neck cancer. However, to maintain its effectiveness with the lowest possible toxicity, the fractionation of radiation doses has been investigated as an option to conventional radiotherapy^9^, considering that the use of different fractions (lower or higher than conventional) could trigger fewer adverse effects and potentiate therapeutic effects, such as better local control and tissue tolerability^9^ ^,^ ^10^. Hypofractionation (a fractionated radiotherapy modality) has been investigated as a modality to treat head and neck cancer, such as early-stage laryngeal cancer, as well as palliative radiotherapy^10^. On the other hand, benefits and toxicities related to this type of fractionation are still being investigated^9^ ^,^ ^10^.

Based on this state-of-the-art, considering that the previous studies only compared the activation of gelatinases after a conventional radiotherapy protocol ^2^ ^,^ ^3^, a question arose: could hypofractionation influence the activity of gelatinases in DEJ? Therefore, the objective of this study was to compare the activation of gelatinases in dentin-enamel junction and underlying dentin of permanent teeth after experimental radiotherapy in conventional and hypofractionated modalities. The investigated hypothesis (H_1_) was that hypofractionated radiotherapy modality could reduce the activity of gelatinases when compared to conventional.

## Materials and methods

### Study design

This was an *in vitro* study with human teeth (third molars), obtained from the biobank of the School of Dentistry of Ribeirão Preto - University of São Paulo (FORP/USP) and approved by the Research Ethics Committee of FORP/USP (CAAE: 38189920.5.0000.5419). To allow the analyses, 15 human teeth were needed. The treatment factor, represented by the experimental radiotherapy protocol, was divided into three groups: control (non-irradiated), conventional and hypofractionated. The tooth region factor, represented by DEJ and underlying dentin areas, was divided into three levels: cervical, cuspal, and pit regions. Thus, each type of treatment received ten tooth slices (*n* = 10). All slices had the same tooth regions.

### Teeth selection and storage

Recently extracted third molars were eligible. The teeth were stored in distilled water (4ºC) and cleaned with pumice paste and water, using a Robinson brush (MK Life, Porto Alegre, Brazil) at low speed ^11^ ^,^ ^12^. After prophylaxis, each selected tooth was tactilely and visually inspected using an exploratory probe and stereomicroscope (Nikon Instrument Inc., Melville, USA) at 10-x magnification. Only intact third molars, which did not present fractures, cracks, injuries, or defects, were eligible. The selected teeth were stored in a supersaturated thymol solution (0.1%) for one week. Then, the teeth were washed in running water and stored in distilled water (4°C) until the beginning of the experimental radiotherapy protocols.

### *In vitro* exposure to experimental radiotherapy protocols 

To expose the teeth to experimental radiotherapy protocols, 21-well plastic boxes were used (Hidraveda^®^, Araçatuba, Brazil). The teeth were positioned and aligned to obtain a uniform exposure, ensuring that all received doses of ionizing radiation evenly (400UM per minute). The source of the ionizing radiation was a linear energy accelerator (Elekta AB, Stockholm, Sweden). Internal control was performed with a nanoDot™ dosimeter (Landauer Inc., Glenwood, USA). During exposures, each box was filled with distilled water (10ml), completely covering the teeth to keep them moist. At the end of each experimental cycle, the teeth were immersed in artificial saliva and kept in a kiln (37ºC) until the next cycle ^11^ ^,^ ^13^. In conventional radiotherapy protocol, standard fractionation was used with a total dose of ionizing radiation of 70Gy, divided into 35 fractions of 2Gy per day. Exposure took place on five consecutive days for seven weeks. In the hypofractionated radiotherapy protocol, the total dose was set at 55Gy, divided into 20 fractions of 2.75Gy per day. The exposure took place on five consecutive days for four weeks ^9^ ^,^ ^14^ ^,^ ^15^.

### *In situ* zymography 

After each experimental protocol, the teeth were sectioned 1mm below the cementoenamel junction using a Minitom cutting machine (Struers A/S, Copenhagen, Denmark) under refrigeration, and the crowns were sectioned from the mesial to distal surface at a thickness of 0.6mm using an IsoMet™ 1000 cutting machine (Buehler Ltd., Lake Bluff, USA) ^12^. The tooth slices were immersed in 1mg/ml of sodium borohydride solution (Sigma-Aldrich^®^, Saint Louis, USA) for three 15-minute intervals and washed in phosphate-buffered saline (PBS). Then, the incubation of a gelatinous substrate associated with fluorescein isothiocyanate (DQ™ Gelatin; Molecular Probes, Eugene, USA) dissolved in PBS at a concentration of 1mg/ml (37ºC) was carried out. The incubation lasted three hours and was carried out in a dark and humidified chamber ^2^ ^,^ ^3^. As a positive control, tooth slices were pre-incubated with 20 mM ethylenediaminetetraacetate (EDTA; Sigma-Aldrich^®^, Saint Louis, USA) and then EDTA was added to the gelatinous substrate ^2^ ^,^ ^3^.

**Figure 1:**
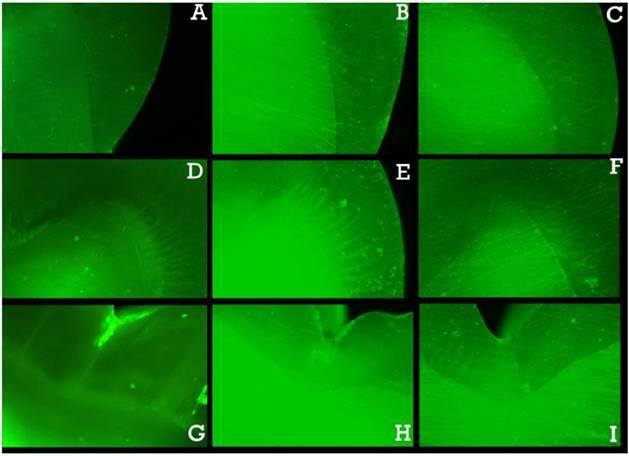
Microscopic images after *in situ* zymography in the cervical region (A: control, B: conventional, and C: hypofractionated), in the cusp tip region (D: control, E: conventional, and F: hypofractionated), and in the pit region (G: control, H: conventional and I: hypofractionated).

**Figure 2:**
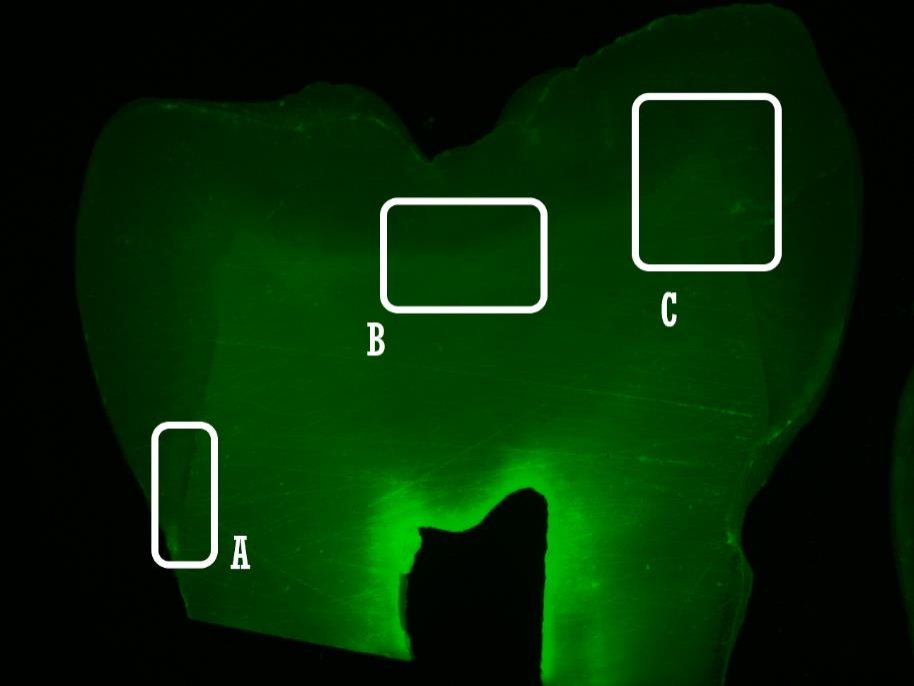
Microscopic images after *in situ* zymography representing the evaluated tooth regions: cervical (A), pit (B), and cuspal (C).

The sections were evaluated in a fluorescence microscope at 10-× magnification, using the Alexa Fluor 43HE filter (FT570, BP550/25, BP605/70, Carl Zeiss, Germany). Photographs of each slice were evaluated by optical densitometry using Image J. software (National Institutes of Health, Bethesda, USA). To quantify the fluorescence intensity, areas of interest were established, as shown in Figures 1 and 2. Both in DEJ and underlying dentin, the cervical, cuspal, and pit regions were evaluated. In the software, the fluorescence intensity was obtained in arbitrary fluorescence units (mm^2^) in each predetermined area. The selection of areas of interest was made by the same operator ^2^ ^,^ ^3^.

### Statistical analyses

Statistical analyses were performed using the JAMOVI software (version 1.6.16, Sydney, Australia). Data normality was assessed in distribution graphs and using the Shapiro-Wilk test. Considering a non-normal distribution, the non-parametric Kruskal-Wallis test was used to compare the fluorescence intensity among independent experimental groups, according to the radiotherapy protocol, followed by the Dwass-Steel-Critchlow-Fligner test (DSCF) as a *post hoc* test in pairwise comparisons. The results were represented in the tables by the median and by the first (Q1) and third (Q3) quartiles, in addition to the interquartile range (IQR).

The Wilcoxon test was used to compare the fluorescence intensity between DEJ and underlying dentin within each experimental radiotherapy group, without considering the tooth regions. Spearman's *rho* correlation coefficient (ρ) was used to assess the correlation between fluorescence intensities in DEJ and underlying dentin among the experimental radiotherapy groups (regardless of the tooth regions). The Friedman test was used to compare the fluorescence intensity among tooth regions (cervical, cuspal, and pit) within each experimental radiotherapy group, both in DEJ and underlying dentin. *Post hoc* pairwise comparisons were performed using the Durbin-Conover test. The significance level was set at 5% (α = 0.05).

## Results

Table 1 shows the results of *in situ* zymography in DEJ and underlying dentin by optical densitometry, considering all tooth regions. Both in DEJ and underlying dentin, statistically significant differences were observed among all experimental groups (*p* <0.001). A higher fluorescence intensity was observed in the group exposed to conventional radiotherapy protocol (CR), followed by the group exposed to hypofractionated (HR) and the control group. In addition, in all experimental groups, the fluorescence intensity was statistically lower in DEJ when compared to underlying dentin (all *p* <0.001).

**Table 1:** *In situ* zymography analyses in dentin-enamel junction and underlying dentin.

Structure		Experimental groups (radiotherapy protocol)		
		Control	Conventional	Hypofractionated
Dentin-enamel junction	Median	36.0^A^	58.2^B^	51.2^C^
	Q1	34.2	52.0	41.4
	Q3	44.8	61.3	56.2
	IQR	10.6	9.3	14.8
Underlying dentin	Median	43.9^A^	66.0^B^	59.6^C^
	Q1	36.2	64.4	52.7
	Q3	49.7	67.0	64.1
	IQR	13.5	2.6	11.4
Dentin-enamel junction *versus* Underlying dentin		*p* < 0.001	*p* < 0.001	*p* < 0.001

A/B/C: statistically significant differences in fluorescence intensity (arbitrary fluorescence units; mm^2^) among experimental radiotherapy groups.

Table 2 shows the results of *in situ* zymography in DEJ for each tooth region evaluated: cervical, cuspal, and pit. In the cervical region, statistically significant differences were observed among experimental groups (*p* = 0.003). The fluorescence intensity of the control group was significantly lower than the CR (*p* = 0.006) and HR (*p* = 0.041) groups, which did not differ statistically from each other (*p* = 0.448). In the cuspal region, statistically significant differences were also observed among experimental groups (*p* = 0.001). The fluorescence intensity of the control group was significantly lower than the CR group (*p* = 0.003). However, no statistically significant difference was observed between the HR group when compared to CR (*p* = 0.131) or control (*p* = 0.055) groups. In the pit region, statistically significant differences were also observed among experimental groups (*p* <0.001). The fluorescence intensity of the control group was significantly lower than the CR (*p* <0.001) and HR (*p* = 0.003) groups, which did not differ statistically from each other (*p* = 0.220). In addition, tooth slices incubated with EDTA showed reduced enzymatic activity, indicating that gelatinolytic degradation was due to MMP activation.

**Table 2:** *In situ*, zymography analyses in the dentin-enamel junction of each tooth region evaluated: cervical, cuspal, and pit.

Tooth region (dentin-enamel junction)		Experimental groups (radiotherapy protocol)		
		Control	Conventional	Hypofractionated
Cervical	Median	34.8^A/D^	53.6^B/D^	48.0^B/DE^
	Q1	33.9	45.6	39.7
	Q3	39.7	58.1	50.7
	IQR	5.8	12.5	11
Cuspal	Median	35.6^A/D^	56.4^B/D^	42.9^AB/D^
	Q1	34.1	52.5	39.2
	Q3	36.4	60.3	55.0
	IQR	2.3	7.8	15.8
Pit	Median	44.1^A/D^	60.9^B/E^	55.2^B/E^
	Q1	37.1	59.4	52.9
	Q3	46.8	63.0	59.3
	IQR	9.7	3.6	6.4
Cervical *versus* Cuspal *versus* Pit		*p* = 0.150	*p* = 0.006	*p* = 0.045

A/B/C: statistically significant differences in fluorescence intensity (arbitrary fluorescence units in mm^2^) among experimental radiotherapy groups. D/E: statistically significant differences in fluorescence intensity (arbitrary fluorescence units; mm^2^) among tooth regions within each experimental radiotherapy group.

Table 2 also shows the comparison of fluorescence intensity in DEJ among tooth regions (cervical, cuspal, and pit) in each experimental group. In the control group, no statistically significant difference was observed in the fluorescence intensity among tooth regions (*p* = 0.150). In the CR group, the fluorescence intensity in the pit region was significantly higher when compared to cervical (*p* <0.001) and cuspal (*p* = 0.007) regions, which did not differ statistically from each other (*p* = 0.223). In the HR group, the fluorescence intensity in the pit region was significantly higher when compared to the cuspal region (*p* = 0.012). No statistically significant difference was observed between cervical and cuspal (*p* = 0.321) and cervical and pit (*p* = 0.091) regions.

Table 3 shows the results of *in situ* zymography in dentin underlying DEJ for each tooth region evaluated: cervical, cuspal, and pit. In the cervical region, statistically significant differences were observed among experimental groups (*p* <0.001). The fluorescence intensity of the control group was significantly lower than the CR (*p* = 0.003) and HR (*p* = 0.006) groups, which did not differ statistically from each other (*p* = 0.730). In the cuspal region, statistically significant differences were observed among experimental groups (*p* <0.001). The fluorescence intensity of the control group was significantly lower than the CR (*p* = 0.001) and HR (*p* = 0.018) groups, which did not differ statistically from each other (*p* = 0.060). In the pit region, statistically significant differences were also observed among all groups (*p* <0.001).

**Table 3:** *In situ*, zymography analyses in underlying dentin of each tooth region evaluated: cervical, cuspal, and pit.

Tooth region (underlying dentin)		Experimental groups (radiotherapy protocol)		
		Control	Conventional	Hypofractionated
Cervical	Median	38.4^A/D^	62.0^B/D^	57.5^B/D^
	Q1	34.7	52.3	48.9
	Q3	47.3	66.4	59.6
	IQR	12.6	14.1	10.7
Cuspal	Median	43.4^A/D^	66.6^B/D^	56.3^B/D^
	Q1	39.2	65.2	54.9
	Q3	49.5	67.7	65.0
	IQR	10.3	2.5	10.1
Pit	Median	48.8^A/D^	66.6^B/D^	62.1^C/D^
	Q1	40.3	65.4	60.9
	Q3	53.1	67.0	63.3
	IQR	12.1	1.6	2.4
Cervical *versus* Cuspal *versus* Pit		*p* = 0.067	*p* = 0.061	*p* = 0.741

Table 3 also shows the comparison of fluorescence intensity in dentin underlying DEJ among tooth regions (cervical, cuspal, and pit) in each experimental group. There were no statistically significant differences in fluorescence intensity among tooth regions in the control (*p* = 0.067), CR (*p* = 0.061), and HR (*p* = 0.741) groups.

Table 4 shows the correlations between fluorescence intensities in DEJ and underlying dentin (considering all tooth regions). A significant, positive, and strong correlation was observed between fluorescence intensities in the control and HR group, while a significant, positive, and moderate correlation was observed in the CR group.

**Table 4:** Correlations between fluorescence intensities of dentin-enamel junction and underlying dentin, considering all tooth regions.

Correlation		Dentin-enamel junction		
		Control	Conventional	Hypofractionated
Underlying dentin	Control	*p* < 0.001 ρ = 0.870	-	-
	Conventional	-	*p* < 0.001 ρ = 0.685	-
	Hypofractionated	-	-	*p* < 0.001 ρ = 0.703

## Discussion

This study compared the activation of gelatinases in DEJ and underlying dentin of permanent teeth after experimental radiotherapy in conventional and hypofractionated modalities. The hypothesis investigated (H_1_) was partially accepted. Disregarding the evaluation of tooth regions (general analysis), the exposure to hypofractionated radiotherapy protocol induced a lower activation of gelatinases when compared to conventional. However, in analyses by tooth regions, significant statistical differences between hypofractionated and conventional radiotherapy protocols remained only in the underlying dentin of the pit region, although the fluorescence intensity presented higher values in the conventional group in all regions, both in DEJ and underlying dentin.

It was possible to observe that intragroup variabilities affected comparisons by region (10 *versus* 10), while the general analysis (30 *versus* 30) made the intergroup difference significant, despite intragroup variabilities. Moreover, except for the cuspal region in DEJ, the activation of gelatinases was consistently higher in groups exposed to experimental radiotherapy when compared to the control group, both in conventional and hypofractionated modalities. In the cuspal region, the activation of gelatinases in the DEJ did not show significant differences between the control group and the hypofractionated protocol (which showed the greatest variability between the subgroups).

None of the previous investigations on the activation of gelatinases after radiotherapy exposure investigated an alternative modality to conventional protocols and this is the main differential of this approach ^2^ ^,^ ^3^ ^,^ ^5^ ^,^ ^16^. Radiotherapy modalities are important strategies for controlling and curing head and neck cancer. However, they must be chosen taking into account therapeutic potential and associated toxicities, considering better survival rates and quality of life during cancer treatment ^17^ ^,^ ^18^. Considering the principles of radiobiology, hypofractionation is based on the delivery of higher fractions in a smaller number of sessions, enabling the delivery of the ionizing radiation dose planned. Hypofractionated radiotherapy can be an interesting strategy for frail patients, allowing better control of symptoms while acting on tumor control. However, the risk of late toxicity may be a concern. Although the use of hypofractionated radiotherapy in HNC is relevant, especially in palliative care ^19^ ^,^ ^20^, the side effects of this modality on dental hard tissues and oral health are still poorly explored.

For other types of cancer, such as breast and prostate neoplasms, there is evidence of the non-inferiority of the hypofractionated modality when compared to conventional fractionation, considering local control and long-term toxicities. Hypofractionation can also modify treatment duration and positively impact radiotherapy service availability and treatment-related costs ^21^. Otherwise, in HNC, hypofractionated modality is often used for palliative treatment, although there is evidence for its adjuvant use, which is still under investigation ^22^. Then, this study contributes to understanding the oral toxicities related to this treatment modality.

Exploring the outcomes described here, it is necessary to recognize that these results are linked to experimental exposure of tooth slices (*in vitro* radiotherapy). Indeed, obtaining sound teeth from patients with head and neck cancer is challenging. However, a previous study did not observe differences in gelatinolytic activity between *in vivo* and *in vitro* tooth irradiation, as well as a higher activity in dentin when irradiated and non-irradiated teeth were compared, corroborating the outcomes described here on the effect of radiotherapy on gelatinolytic activity ^16^. On the other hand, another previous study did not observe the same outcome and *in vivo* exposure to radiotherapy protocol was not associated with the activation of gelatinases. However, it is important to consider that this study used different dental elements, some of which were decayed or restored ^5^. Gelatinolytic enzymes, MMP-2 and MMP-9, may be related to the progression of caries disease and integrity of hybrid layer in dentin after adhesive restorations, as well as the concentration of MMP-2 in dentin close to DEJ of non-irradiated teeth can also be observed, demonstrating that gelatinases may be physiologically present in this region ^23^ ^,^ ^24^.

Moreover, RRC has an accelerated course, considered an aggressive pattern of the disease. Along with the activity of gelatinases, structural changes caused by ionizing radiation, such as reductions of microhardness values, can make tooth elements susceptible to caries disease ^25^ ^,^ ^26^. Regarding the role of gelatinases, it was demonstrated here that activation in underlying dentin was significantly higher when compared to DEJ. Indeed, it is possible to understand that the initial damage occurs in DEJ. However, when reaching dentin, the higher activation of gelatinases can contribute to the rapid progression of carious lesions ^25^ ^,^ ^26^. Nonetheless, it was also demonstrated a correlation between the activation of gelatinases in DEJ and underlying dentin, even if in greater intensity in the latter.

This outcome supports the hypothesis that activation of gelatinases in underlying dentin may be important in the progression of carious lesions, considering that higher activity in DEJ suggests higher activity in the underlying dentin. Although the presence of gelatinases in this region is physiological ^23^, exposure to ionizing radiation significantly increased their activity. It is worth pointing out that the loss of protein content in dental tissues, such as collagen fibers, can influence the mechanical properties of dental tissues, contributing to structural loss and the occurrence of carious lesions. This brings us to the role of gelatinases in this process ^25^ ^,^ ^26^. The outcomes in this study, added to those described in prior studies ^2^ ^,^ ^3^, allow us to understand that gelatinases are present in the organic matrix of dental hard tissues, such as enamel and dentin, as well as the exposure to radiotherapy can increase the activation of these enzymes, both in DEJ and underlying dentin.

Interestingly, although it is described that the appearance of RRC is in the cervical and cuspal or incisal regions, instead of pits ^25^ ^,^ ^26^, it was demonstrated here that the activation of gelatinases in the pit region was not statistically inferior. The exposure to conventional radiotherapy protocols increased the activity of gelatinases in the underlying dentin of pit regions when compared to hypofractionated. It is not possible to infer the clinical impact of radiotherapy modalities on the activation of gelatinases and a higher activity in the pit region may not translate into caries in this tooth region. First, the occurrence of caries in pits is common in general populations, but biofilm retention in this region is a key feature. Although it is possible to observe depth pits, which favor biofilm retention, there are low mechanical chewing efforts ^27^ ^,^ ^28^. Thus, it is possible to hypothesize that changes related to ionizing radiation exposure in the pit region are not related to mechanical stress to trigger structural delamination. Hence, dental caries in pit regions could not be clinically associated with radiotherapy treatment, despite the gelatinolytic activity demonstrated here. However, this hypothesis needs systematic investigation.

Lastly, it is important to recognize that the impact of radiotherapy on the oral cavity, including the activation of gelatinases and the development of RCC, occurs simultaneously with other events, such as hyposalivation (reduced salivary flow) and changes in oral microbiota. In addition, it is possible to observe changes in carbohydrate intake (increase) and difficulties in maintaining proper oral hygiene habits during the treatment of HNC ^6^. Moreover, structural changes such as microhardness reduction can be added to this list ^7^ ^,^ ^11^. All these events occur simultaneously and translate into the risk of dental caries after radiotherapy experienced by HNC patients exposed to ionizing radiation ^8^.

Although the *in vitro* approach is the first step to clinically understand the impact of ionizing radiation on dental hard tissues, it is important to consider these events in the occurrence of oral diseases during radiotherapy treatment, especially RCC. Hence, these findings may contribute to future perspectives in this research area, but it is possible to hypothesize that other phenomena that cannot be reproduced *in vitro* clinically modify the outcomes presented here, which also requires systematic investigations. In addition, the reduced number of slices in the secondary analysis (by tooth regions) is an important limitation of this study.

## Conclusion

It is possible to conclude that radiotherapy protocols increased the activity of gelatinases in dentin-enamel junction and underlying dentin. The hypofractionated protocol showed a lower activation than conventional in general analyses, but this difference was not observed in analyses of tooth regions.
